# Single-cell dissection, hdWGCNA and deep learning reveal the role of oxidatively stressed plasma cells in ulcerative colitis: Oxidative stress in plasma cells and ulcerative colitis

**DOI:** 10.3724/abbs.2023237

**Published:** 2023-10-09

**Authors:** Shaocong Mo, Xin Shen, Baoxiang Huang, Yulin Wang, Lingxi Lin, Qiuming Chen, Meilin Weng, Takehito Sugasawa, Wenchao Gu, Yoshito Tsushima, Takahito Nakajima

**Affiliations:** 1 Department of Digestive Diseases Huashan Hospital Fudan University Shanghai 200040 China; 2 Guangdong Medical University Dongguan 523808 China; 3 Department of Nephrology Zhongshan Hospital Fudan University Shanghai 200032 China; 4 Department of Thoracic Surgery The First Affiliated Hospital College of Medicine Zhejiang University Hangzhou 310003 China; 5 Department of Anesthesiology Zhongshan Hospital Fudan University Shanghai 200032 China; 6 Laboratory of Clinical Examination and Sports Medicine Department of Clinical Medicine Faculty of Medicine University of Tsukuba Ibaraki 305-8577 Japan; 7 Department of Diagnostic and Interventional Radiology University of Tsukuba Ibaraki 305-8577 Japan; 8 Department of Diagnostic Radiology and Nuclear Medicine Gunma University Graduate School of Medicine Maebashi 371-8511 Japan

**Keywords:** single-cell, hdWGCNA, deep learning, plasma cell, oxidative stress

## Abstract

Ulcerative colitis (UC) develops as a result of complex interactions between various cell types in the mucosal microenvironment. In this study, we aim to elucidate the pathogenesis of ulcerative colitis at the single-cell level and unveil its clinical significance. Using single-cell RNA sequencing and high-dimensional weighted gene co-expression network analysis, we identify a subpopulation of plasma cells (PCs) with significantly increased infiltration in UC colonic mucosa, characterized by pronounced oxidative stress. Combining 10 machine learning approaches, we find that the PC oxidative stress genes accurately distinguish diseased mucosa from normal mucosa (independent external testing AUC=0.991, sensitivity=0.986, specificity=0.909). Using MCPcounter and non-negative matrix factorization, we identify the association between PC oxidative stress genes and immune cell infiltration as well as patient heterogeneity. Spatial transcriptome data is used to verify the infiltration of oxidatively stressed PCs in colitis. Finally, we develop a gene-immune convolutional neural network deep learning model to diagnose UC mucosa in different cohorts (independent external testing AUC=0.984, sensitivity=95.9%, specificity=100%). Our work sheds light on the key pathogenic cell subpopulations in UC and is essential for the development of future clinical disease diagnostic tools.

## Introduction

Ulcerative colitis (UC) is a chronic, idiopathic, inflammatory disease that affects the colonic mucosa [ 1, 2] . The incidence and prevalence of UC are increasing every year, especially in developing countries [ 3, 4] . UC is challenging to cure entirely, and patients often require long-term treatment [5]. Furthermore, persistent UC can increase the risk of colorectal cancer [6]. However, there is a lack of diagnostic methods for UC other than endoscopic biopsy pathology [7]. A delayed diagnosis of UC significantly increases the risk of surgical interventions and other potentially life-threatening complications [8]. Thus, it is crucial to identify reliable diagnostic markers for the early detection of UC and to develop new molecular stratification techniques to guide personalized treatment of UC patients.

Mechanistically, the development of UC can be resulted from synergistic interactions of multiple epithelial, immune, and stromal cells. Dysfunctions of these cells might contribute to the disease [9]. A large number of studies have reported that the pathogenesis of UC is associated with various immune cell types, such as macrophages [ 10, 11] , innate lymphocytes [ 12‒ 14] and CD 4 T cell subsets [ 15, 16] . Furthermore, it was revealed that the composition of CD 8 T cells is widely heterogeneous and that UC-associated CD8 effector T cells could reduce the regulatory function of excessive inflammation by producing tumor necrosis [17]. Heterogeneity of plasma cells was also believed to be linked to the progression and outcome of UC [18]. It has been demonstrated that there is a highly dysregulated B-cell response in UC [19]. This evidence illustrates that exploring the molecular mechanisms and functions of cellular subpopulations in the UC microenvironment could provide strategies for potential new biomarkers.

In this work, we found a significant increase in plasma cell subpopulations in UC patients by single-cell sequencing analysis. We identified specific PC gene modules by high-dimensional weighted gene co-expression network analysis (hdWGCNA). Protein‒protein interaction (PPI) network analysis and enrichment analysis showed that the upregulated plasma cell subpopulation is mainly associated with oxidative stress. PC oxidative stress genes facilitate UC diagnosis *via* machine learning and are associated with multiple immune cell infiltrations. We also verified the existence of oxidatively stressed PCs in colitis through spatial transcriptomics. Finally, we synthesized photographs of 11 key genes and immune cells to establish a gene-immune convolutional neural network deep learning model for the diagnosis of UC.

## Materials and Methods

### Data acquisition

All UC mucosa datasets were screened in the Gene Expression Omnibus (GEO) database ( http://www.ncbi.nlm.nih.gov/geo/), including single-cell sequencing data GSE182270 [19], microarray data GSE87466 [20] and GSE87466 [21], RNA-sequencing data GSE165512 and spatial transcriptomic data GSE190595 [22].

### Single-cell sequencing data processing and high-dimensional WGCNA (hdWCNA)

Cells expressing between 200 and 10,000 genes were identified. A 20% mitochondrial proportion gene was also set as a threshold for filtering. The top 2000 highly variable genes were identified for scaling with the *FindVariableFeature* function in the R package Seurat [23]. *FindNeighbors* and *FindCluster* were subsequently applied to obtain cell clusters. Next, the single-cell dataset was annotated with known cell markers [24]. To further identify specific subpopulations of plasma cells in UC patients, the plasma cells were redimensionalized and reclustered. For WGCNA of the single-cell sequencing data, the hdWGCNA package was used according to the standard pipeline of hdWGCNA [ 25, 26] .

### Pseudotime analysis and cell–cell interaction analysis

Plasma cells were extracted for subsequent pseudotime analysis. The cell differentiation state type was determined using the *DDRtree* method and the *reduceDimension* function in the monocle package. Then, we used the *plot_cell_trajectory* function to visualize the differentiation trajectory of cells [ 27, 28] . To study intercellular interactions mediated by ligand‒receptor complexes, the CellChat package was used [29]. The major signal inputs and outputs in all UC cell subpopulations were assessed by CellChatDB. Human on cellchat [ 29, 30] .

### Protein‒protein interaction analysis and enrichment analysis

The GeneMANIA data resource ( http://www.genemania.org) was used to further identify potential interaction networks between target module proteins [ 31, 32] . The nodes represent proteins, and the edges represent the interaction between two proteins. Functional enrichment was performed using the clusterProfiler package [33] and Metascape online tool [34].

### Non-negative matrix factorization (NMF), gene set scoring and immune infiltration analysis

The NMF algorithm was used to subclass patients based on RNA-seq bulk gene expression profiles [ 35‒ 37] . The optimized cluster number was selected by cophenetic value. Scores for plasma cell subpopulations characterized by oxidative stress were calculated using single sample gene set enrichment analysis (ssGSEA) [38]. To further reveal the relationship between UC and the immune microenvironment, MCPcounter [39] was utilized to assess the infiltration abundance of 10 immune cell species.

### Spatial transcriptomic analysis

For spatial transcriptomic data, the Seurat pipeline was utilized. Mitochondrial and ribosomal genes were filtered, and genes expressing fewer than 10 spots were filtered [40]. Then, the expression profile underwent SCTransformed. SpatialDimPlot and SpatialFeaturePlot were used to visualize the landscape of the section. Cd138 was selected as the marker for plasma cells, and a signature of the response to oxidative stress was obtained from the Gene Ontology database. Gene set evaluation was performed using the AddModuleScore function [30].

### Machine learning and deep learning

Univariate logistic regression was utilized to identify key diagnostic genes in plasma cells characterized by oxidative stress. Subsequently, the Least Absolute Shrinkage and Selection Operator (LASSO) was employed to further filter the variables [41]. To establish models with high accuracy and stable performance, the models were built by the mlr3 package [42]. The best model was selected among 10 machine learning models, including k-nearest neighbor (KNN), linear discriminant analysis (LDA), logistic regression (LR), multinomial logit model (multinorm), naïve Bayes (NB), quadratic discriminant analysis (QDA), random forest (RF), recursive partitioning and regression trees (RPART), support vector machine (SVM) and extreme gradient boosting (XGBoost). Finally, to validate the accuracy of the machine learning results and the relationship between the target genes and immune cells, 11 key genes and 10 immune cells were synthesized into images for deep learning (N _j,I_=immune _i_ /gene _j_). A deep learning model based on the Keras and TensorFlow frameworks using convolutional neural networks (CNNs) was constructed.

### Statistical analysis

The Wilcoxon test was used to make comparisons between the two groups. The Kruskal‒Wallis test was used to make comparisons among the three groups. Pearson correlation analysis was used to reveal the relationship between the 11 key genes and 10 types of immune cells. A *P* value<0.05 was considered statistically significant. All analyses were performed in R (version 4.1.3).

## Results

### Altered proportions of plasma cell subpopulations in UC mucosa

**Figure 1:**
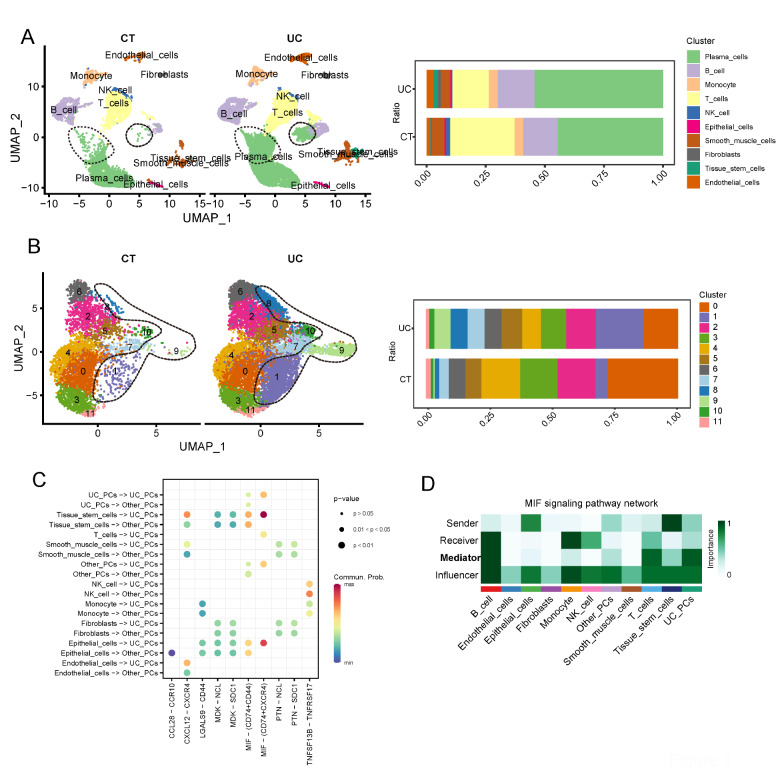
Figure 1 Altered proportions of plasma cell subpopulations in UC mucosa (A) Left panel, plasma cells (PCs) were upregulated in UC (dotted box). Right panel, proportions of each cell type (B) Left panel, clusters 1, 8 and 9 were upregulated in UC (dotted box). Right panel, proportions of each cluster of plasma cells. (C) Cell‒cell interactions between other cell types and PCs. (D) UC PCs acted as mediators of MIF signaling.

To explore the altered cellular composition in UC mucosa compared to normal mucosa, we applied GSE182270 to dissect the cellular composition of UC. After integration by harmony, dimensionality reduction and clustering and cell type annotation, we discovered that the altered proportion of plasma cells (PCs) was most pronounced in UC ( Figure 1A). To further illustrate the specific altered subgroups of PCs, we extracted the PCs and further defined subgroups. It was interesting to find that not all plasma cells were universally up-regulated, but rather Cluster 1, 8 and 9 PCs ( Figure 1B). Additionally, we noted that UC PCs possessed stronger interactions in the MIF pathway than other PCs, receiving MIF signals from T cells, epithelial cells and stem cells ( Figure 1C). Thus, UC PCs were considered to be mediators of the MIF pathway ( Figure 1D).

### hdWGCNA revealed that Cluster 9 is characterized by the blue module

**Figure 2:**
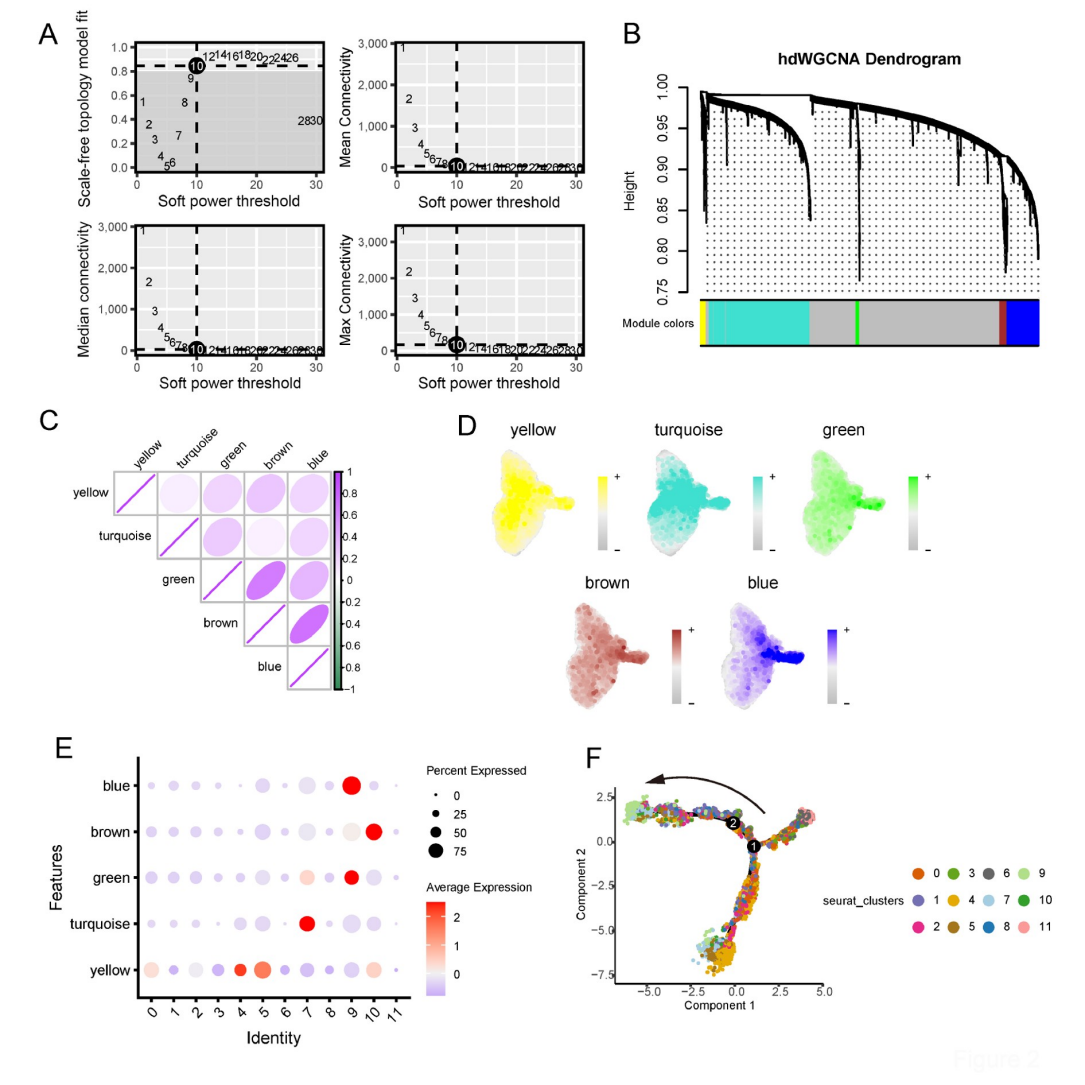
Figure 2 hdWGCNA revealed that Cluster 9 is characterized by the blue module (A) Power value equal to 10 when the network reached a scale-free distribution. (B) Highly variable genes were clustered into 5 modules through hdWGCNA. (C) The correlations between modules. (D) FeaturePlots of different module scores in PCs. (E) Dot plot of the different module scores in PCs. (F) Pseudotime analysis of Cluster 9 featured by the blue module.

To obtain the characteristics of each small subpopulation, we performed hdWGCNA on PCs. We selected a power value of 10 to construct a scale-free network and generated 5 gene modules ( Figure 2A,B). Among the gene modules, blue, brown and green were more inclined to be expressed in clusters 1, 8 and 9 and showed a significant positive correlation ( Figure 2C,D). Significantly, Cluster 9 featured the blue and green modules, and the blue module in particular was the most distinctive considering the feature plot in 2D ( Figure 2D,E and Supplementary Table S1). Furthermore, pseudotime analysis indicated that Cluster 9 is at the end of plasma cell development ( Figure 2F).

### Genes in the blue module participates in oxidative stress

**Figure 3:**
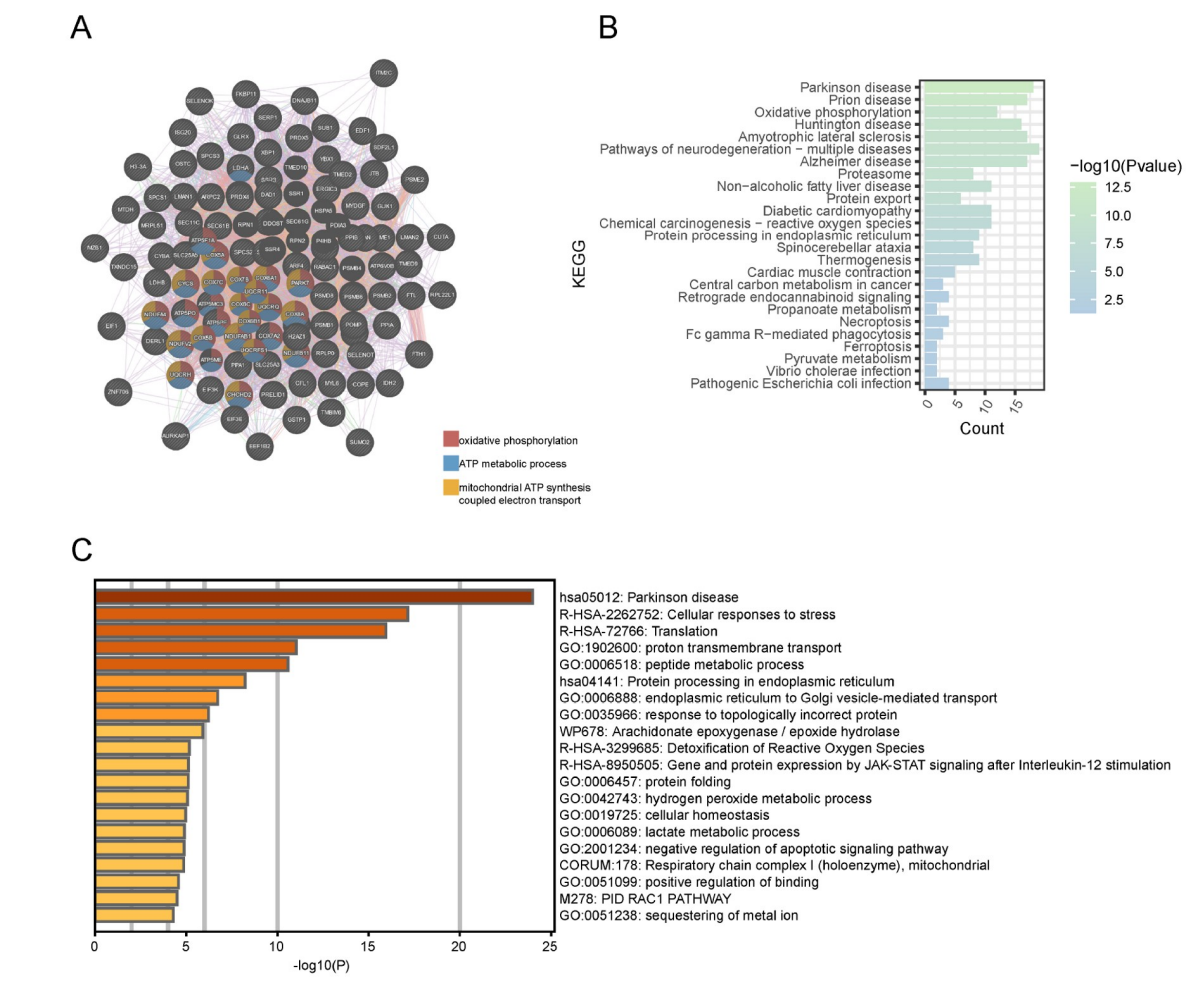
Figure 3 Genes in the blue module participat in oxidative stress (A) Protein‒protein interaction of the genes in the blue module. (B) KEGG enrichment of the genes in the blue module. (C) Functional enrichment of genes in the blue module with Metascape.

To explore the functions of the genes in the blue module, we first conducted protein‒protein interaction (PPI) analysis for the blue module genes. We noticed that the genes at the center of interactions were mainly involved in oxidative phosphorylation, ATP synthesis and metabolic pathways ( Figure 3A). Moreover, KEGG enrichment showed that genes participated in oxidative phosphorylation ( Figure 3B). In addition, we also used Metascape to verify the results, which further implied that Cluster 9 PCs might be those with high levels of oxidative stress ( Figure 3C).

### Machine learning with PC oxidative stress genes

**Figure 4:**
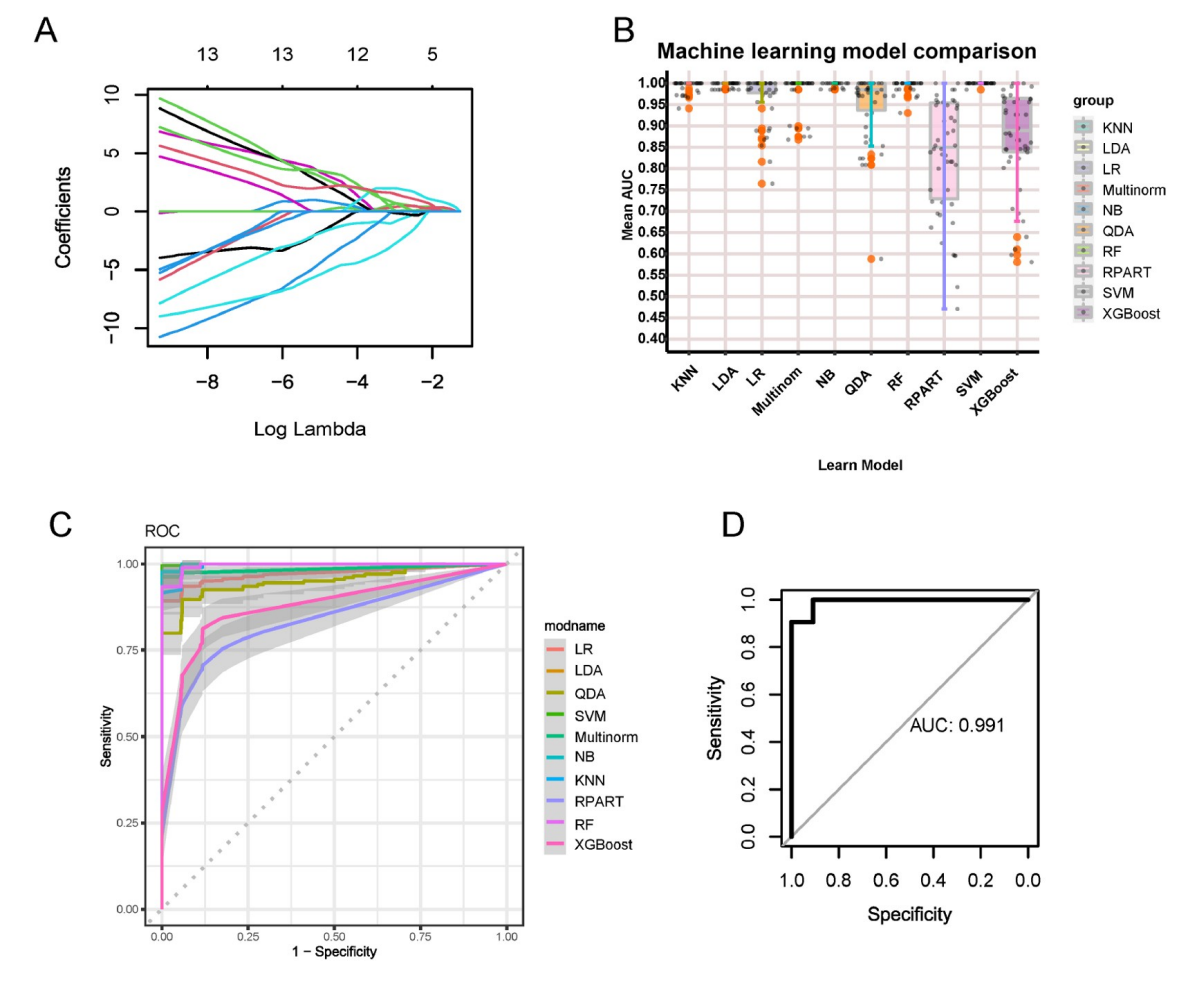
Figure 4 Machine learning with PC oxidative stress genes (A) LASSO regression shrunk the genes to 11. (B) Mean AUC of 5-fold cross-validation for 10 replications of each model. (C) ROC curves and confidence intervals for each model. (D) ROC curve of the model in the independent external validation set.

Considering the presence of oxidatively stressed B cells in the UC mucosa, we sought to use the genes of oxidatively stressed B cells, i.e., the genes of the blue module, for the diagnosis of diseased and normal mucosa. LASSO regression reduced the number of genes to 11 ( Figure 4A and Supplementary Table S2). In integrated machine learning, we incorporated 10 machine learning algorithms, including LDA, SVM, XGBoost, *etc*. In the training set GSE87466, we first conducted 10 repetitions of 5-fold cross-validation to examine the stability of each model. It was found that most of the models possessed good stability. Among the algorithms, RPART and XGBoost performed poorly, while SVM exhibited the best performance ( Figure 4B and Table 1). Additionally, the AUCs of internal validation showed that SVM had the best diagnostic performance ( Figure 4C). Therefore, SVM was applied as the final model and was tested in the external validation set GSE75214, which also performed well (AUC=0.991, sensitivity=0.986, specificity=0.909) ( Figure 4D).

**Table:** **Table 1** Performances of 10 machine learning methods

Method	AUC	Sensitivity	Specificity	FNR	FPR
LR	0.971	0.864	0.980	0.136	0.020
LDA	0.998	0.955	0.985	0.045	0.015
QDA	0.951	0.622	0.992	0.378	0.008
SVM	1.000	0.982	0.991	0.018	0.009
Multinom	0.986	0.926	0.993	0.074	0.007
NB	0.999	1.000	0.965	0.000	0.035
KNN	0.995	0.934	0.975	0.066	0.025
RPART	0.833	0.736	0.910	0.264	0.090
RF	0.996	0.963	0.984	0.037	0.016
XGBoost	0.877	0.737	0.927	0.263	0.073

### PC oxidative stress genes are associated with immune infiltration and available for UC patient subtyping

**Figure 5:**
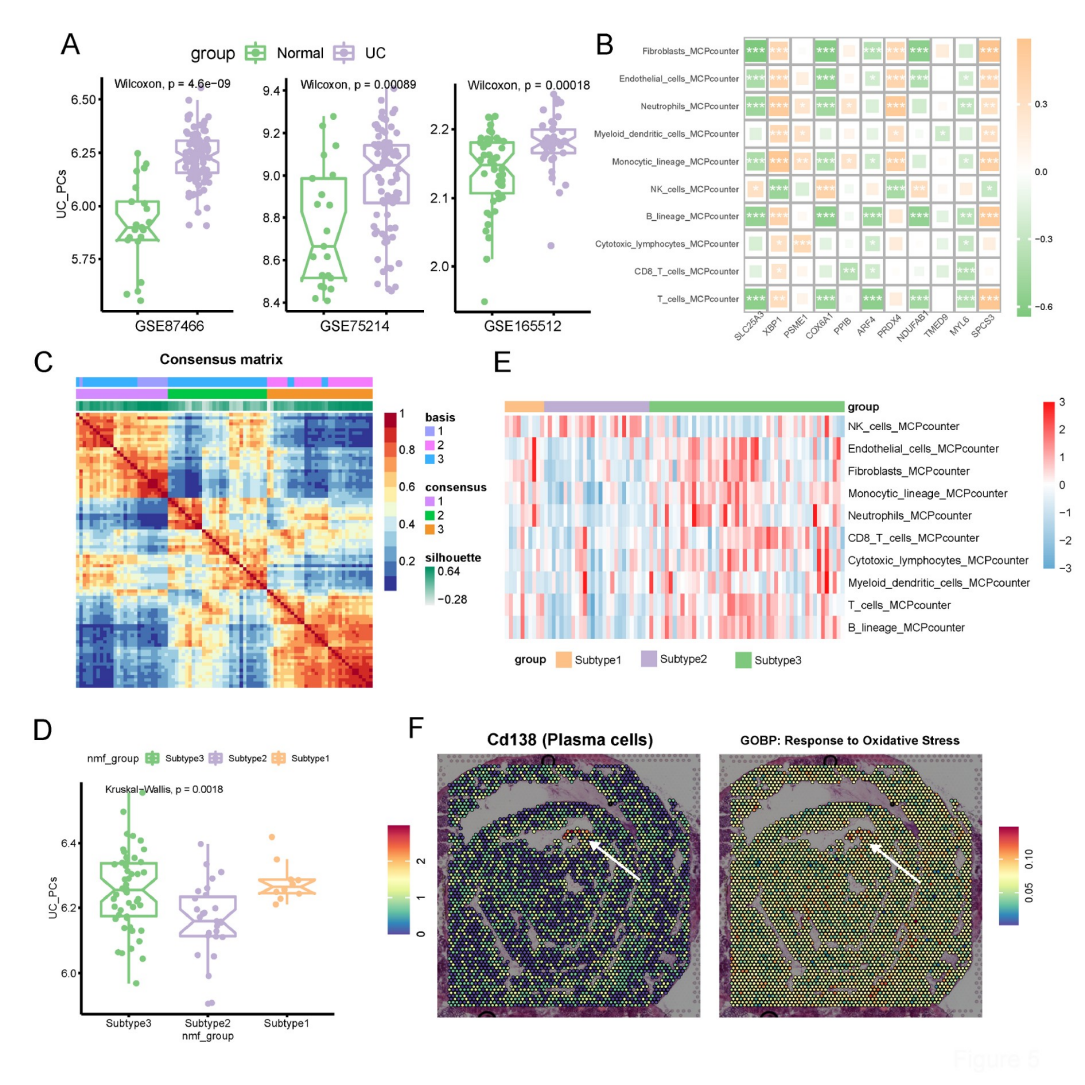
Figure 5 PC oxidative stress genes are associated with immune infiltration and available for UC patient subtyping (A) UC patients did have higher blue module scores than normal mucosa in three independent datasets. (B) Correlations between genes selected by LASSO regression and immune cells. (C) NMF methods subclassed patients into 3 subtypes. (D) Blue module scores of 3 subtypes. (E) Immune infiltration abundance in 3 subtypes. (F) Spatial transcriptomic colocalization of PCs and the oxidative stress signature.

Furthermore, we sought to further explore the relationship of PC oxidative stress genes with immune infiltration and patient heterogeneity at the bulk level. First, we applied ssGSEA to evaluate the abundance of PC oxidative stress genes in normal and UC mucosa of three independent UC datasets, including GSE87466, GSE75214 and GSE165512. The UC mucosa did possess a higher PC oxidative stress score in different datasets ( Figure 5A). Next, we evaluated the immune cell infiltration of the UC mucosa through MCPcounter. It was discovered that hub genes selected by LASSO had strong correlations with immune cells, indicating that PC oxidative stress genes might impact multiple immune cells ( Figure 5B). To reveal the heterogeneity of patients, we clustered patients with PC oxidative stress genes and the NMF algorithm. Patients were classified into 3 subtypes ( Figure 5C). We found that subtypes 1 and 3 had higher levels of immune infiltration and higher PC oxidative stress scores, while subtype 2 had a low level of immune infiltration ( Figure 5D,E). Moreover, spatial transcriptomic data of dextran sulfate sodium (DSS) colitis mice were applied to verify the existence of the specific upregulation of stressed PCs. We found that the infiltration of plasma cells was increased in diseased regions compared with non-lesion regions ( Figure 5F and Supplementary Figure S1, white arrow). Remarkably, there was colocalization of plasma cell abundance and abundance of cellular stress in space, as indicated by the white arrow ( Figure 5F). We thus validated the presence of the aforementioned specificity of oxidatively stressed PCs in colitis.

### Establishment of the gene-immune convolutional neural network model

**Figure 6:**
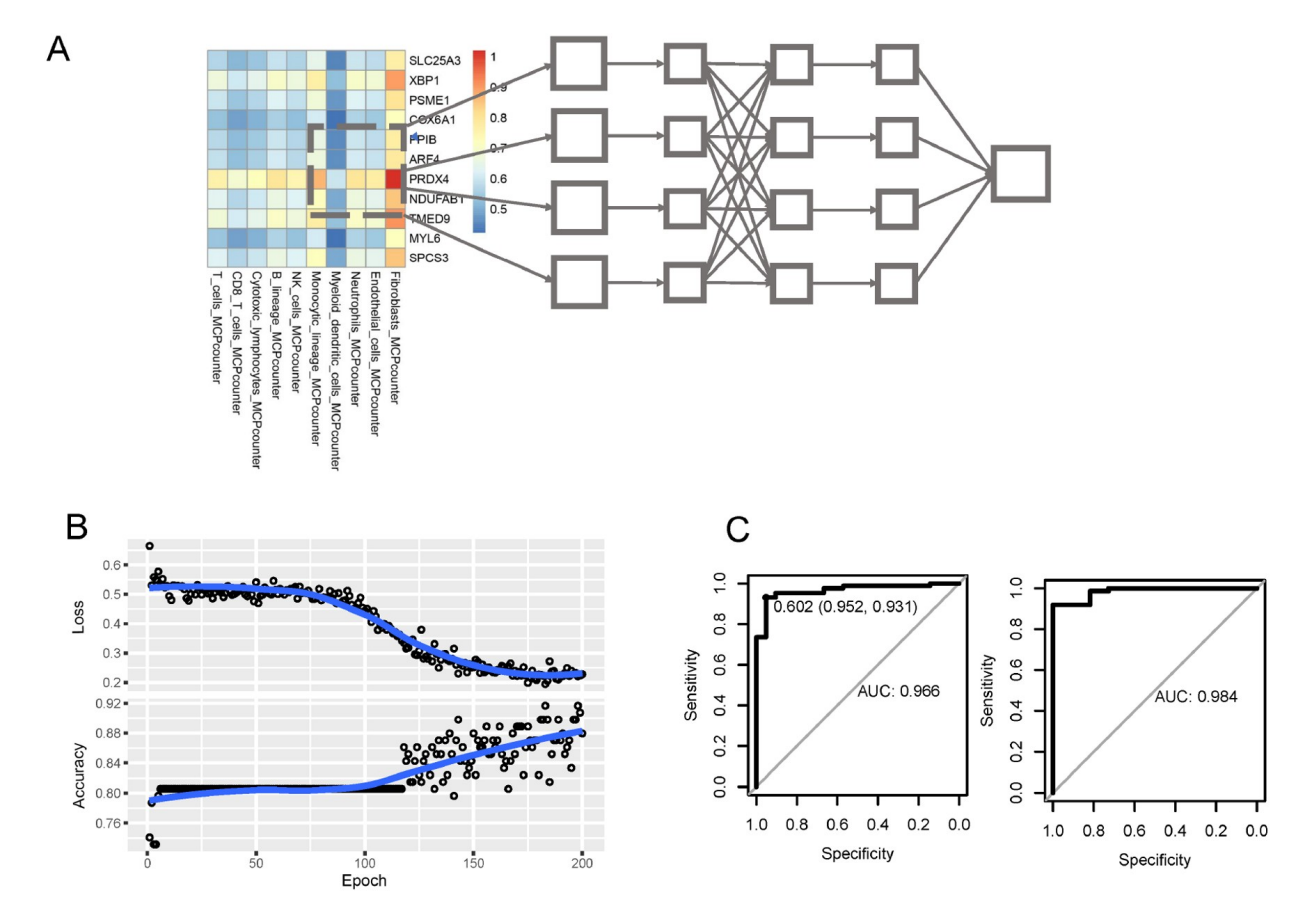
Figure 6 Establishment of the gene-immune CNN model (A) Principle of the model of the gene-immune CNN. (B) Training process of the gene-immune CNN model. (C) Performance of the gene-immune CNN model in the training and validation sets.

Since the important relationship between PC oxidative stress genes and immune cells has been mentioned above, coupled with our need to establish diagnostic techniques independent of the batch effect of the datasets, we developed a gene-immune CNN classifier. In brief, we constructed a gene-immune (11 units long by 10 units wide) heatmap for each patient, with the value of each square being the ratio of the expression of a gene to the infiltration of a particular immune cell in that patient ( Figure 6A). The convolutional neural network was trained using 200 epochs, with GSE87466 as the training set and GSE75214 as the testing set ( Figure 6B). We found that the gene-immune CNN performed well in both the training and testing sets (training AUC=0.602, sensitivity=95.2%, specificity=93.1%, testing AUC=0.984, sensitivity=95.9%, specificity=100%), suggesting its broad application prospects ( Figure 6C).

## Discussion

UC is a chronic gastrointestinal disorder of unknown origin characterized by continuous inflammatory colonic mucosa. Understanding the cellular and molecular mechanisms underlying UC has been a challenge, but recent advances in single-cell RNA sequencing and machine learning have enabled investigation of oxidative stress-related gene-gene and cell-cell interactions in UC. In this study, we used single-cell RNA sequencing and high-dimensional weighted gene co-expression network analysis to identify a disease-specific subgroup of plasma cells characterized by oxidative stress. Additionally, we identified signature genes of oxidatively stressed plasma cells that could be used for disease diagnosis and immune microenvironment analysis.

Plasma cells play a critical role in maintaining intestinal homeostasis, but their involvement in UC remains understudied. A highly dysregulated B-cell response in UC has been previously demonstrated, mainly reflected in the expansion and decreased diversity and maturation of plasma cells [19]. Additionally, it was found that UC is associated with an increase in plasma cells infiltrating colonic tissue [18]. However, few studies have investigated the specific subtypes and corresponding functions of plasma cells associated with UC. Our research emphasizes the role of plasma cells in the pathogenesis of UC. We observed a significant increase in PC abundance and extensive intercellular communication in UC mucosa at the single-cell RNA sequencing level, particularly the subtype characterized by oxidative stress.

Oxidative stress has been recognized as a potential risk factor for UC [43]. Under inflammatory conditions, high production of reactive oxygen species accumulates to generate oxidative stress, which in turn leads to DNA damage, driving the malignant progression of UC [44]. Thus, targeting oxidative stress might provide an exciting avenue to alleviate colonic inflammation, as well as combat inflammation-related DNA damage and subsequent carcinogenesis [ 45, 46] . In this study, oxidative stress-related genes were identified in UC-specific plasma cells by hdWGCNA, which was consistent with the reported correlation between plasma cell aggregation and oxidative stress [47].

B cells are particularly vulnerable to oxidative stress, which can lead to B-cell dysfunction and contribute to the pathogenesis and progression of various diseases, including nonalcoholic fatty liver disease (NAFLD) and hepatocellular carcinoma associated with nonalcoholic steatohepatitis (NASH) [48]. In SLE (systemic lupus erythematosus), oxidative stress in B cells contributes to immune system dysregulation, abnormal activation and processing of cell-death signals, and autoantibody production [49]. In atherosclerosis, oxidative stress leads to the accumulation and dysfunction of B cells in the arterial adventitia layer, thus accelerating atherosclerotic plaque formation [50]. Moreover, increased oxidative metabolism in B cells has been associated with impaired plasma cell differentiation [51]. Taken together, these studies underscore the significance of oxidative stress-related dysfunction of B cells in various disease settings. However, the role of oxidative stress occurring in B cells in UC has not been extensively studied. Our research demonstrated that plasma cells specific to UC were distinguished by a dysregulated oxidative stress gene signature, which could aid in the identification of UC lesions.

The diagnosis of UC is mainly based on clinical presentation and endoscopic findings [52]. The molecular markers used to identify early UC patients are less studied, which is critical for receiving effective treatments. Fortunately, machine learning has been applied to aid the diagnosis of IBD and to predict the risk of relapse and carcinogenesis, with encouraging outcomes [53]. In our study, 11 oxidative stress-related genes were identified in the blue module. *SLC25A3* encodes the mitochondrial phosphate carrier, whose mutation can lead to mitochondrial phosphate-carrier deficiency [54]. *SLC25A3* is also differentially upregulated in UC and plays a potential carcinogenic role in UC-associated colorectal cancer [55]. Specifically, XBP1 is a major endoplasmic reticulum stress-linked transcription factor and has been reported to contribute to cellular resistance to oxidative stress [56]. *XBP1* abnormalities result in intestinal inflammation, thus increasing susceptibility to IBD [57]. PSME1 has been identified as a protein biomarker to signify active IBD using advanced label-free quantification technology for proteomes [58]. *COX6A1* is involved in oxidative phosphorylation and mitochondrial respiration [ 59, 60] , but to date, its engagement in IBD has never been reported. *PPIB* helps to adapt cells to oxidative stress and hypoxic conditions [61] but has not been studied in IBD. *ARF4* has been demonstrated to have anti-apoptotic activity in glioblastoma by inhibiting stress-mediated apoptotic signals [62]. *PRDX4* has been reported to ameliorate lipotoxicity-induced oxidative stress and apoptosis in diabetic cardiomyopathy [63] but has never been studied in the gastrointestinal tract. *NDUFAB1* encodes one of the mitochondrial respiratory chain complexes, which plays an essential role in mitochondrial homeostasis in colitis [64]. *MYL6* is strongly related to increased mitochondrial efficiency, especially under oxidative stress conditions. In summary, almost all the genes in the blue module are strongly related to oxidative stress. Meanwhile, UC mucosa scored significantly higher by the ssGSEA algorithm based on genes in the blue module. Additionally, the expression pattern of blue module genes is closely associated with immune infiltration, indicating their possible role in immunomodulation. To date, endoscopy is currently the gold standard for evaluating UC lesions, but misdiagnosis of UC can occur due to its complex and varied clinical manifestations. Molecular diagnosis based on RNA sequencing of colon tissue has significantly improved diagnostic accuracy [65]. In our study, we demonstrated that our oxidative stress-related signature had stable performance in distinguishing UC lesions through machine learning and deep learning, which could assist in clinical diagnosis and subtype classification and lead to improved UC management.

In conclusion, we successfully identified a disease-specific subgroup of plasma cells characterized by oxidative stress in UC. Our study has established promising machine learning and deep learning models based on PC oxidative stress genes, providing a new paradigm for future UC research.

## Supporting information

Supplementary

## Supplementary Data

Supplementary data is available at *Acta Biochimica et Biophysica Sinica* online.

## References

[1] Høivik ML, Moum B, Solberg IC, Henriksen M, Cvancarova M, Bernklev T (2013). Work disability in inflammatory bowel disease patients 10 years after disease onset: results from the IBSEN Study. Gut.

[2] Torres J, Billioud V, Sachar DB, Peyrin-Biroulet L, Colombel JF (2012). Ulcerative colitis as a progressive disease: the forgotten evidence. Inflammatory Bowel Dis.

[3] Cosnes J, Gower–Rousseau C, Seksik P, Cortot A (2011). Epidemiology and natural history of inflammatory bowel diseases. Gastroenterology.

[4] Loftus EV Jr (2004). Clinical epidemiology of inflammatory bowel disease: incidence, prevalence, and environmental influences. Gastroenterology.

[5] Le Berre C, Ricciuto A, Peyrin-Biroulet L, Turner D (2022). Evolving short- and long-term goals of management of inflammatory bowel diseases: getting it right, making it last. Gastroenterology.

[6] Olén O, Erichsen R, Sachs MC, Pedersen L, Halfvarson J, Askling J, Ekbom A (2020). Colorectal cancer in ulcerative colitis: a Scandinavian population-based cohort study. Lancet.

[7] Dignass A, Eliakim R, Magro F, Maaser C, Chowers Y, Geboes K, Mantzaris G (2012). Second european evidence-based consensus on the diagnosis and management of ulcerative colitis Part 1: definitions and diagnosis. J Crohns Colitis.

[8] Randall J, Singh B, Warren BF, Travis SPL, Mortensen NJ, George BD (2010). Delayed surgery for acute severe colitis is associated with increased risk of postoperative complications. Br J Surg.

[9] Xavier RJ, Podolsky DK (2007). Unravelling the pathogenesis of inflammatory bowel disease. Nature.

[10] Na YR, Stakenborg M, Seok SH, Matteoli G (2019). Macrophages in intestinal inflammation and resolution: a potential therapeutic target in IBD. Nat Rev Gastroenterol Hepatol.

[11] Kamada N, Hisamatsu T, Okamoto S, Chinen H, Kobayashi T, Sato T, Sakuraba A (2008). Unique CD14+ intestinal macrophages contribute to the pathogenesis of Crohn disease via IL-23/IFN-γ axis. J Clin Invest.

[12] Bal SM, Golebski K, Spits H (2020). Plasticity of innate lymphoid cell subsets. Nat Rev Immunol.

[13] Lim AI, Menegatti S, Bustamante J, Le Bourhis L, Allez M, Rogge L, Casanova JL (2016). IL-12 drives functional plasticity of human group 2 innate lymphoid cells. J Exp Med.

[14] Zhou L, Chu C, Teng F, Bessman NJ, Goc J, Santosa EK, Putzel GG (2019). Innate lymphoid cells support regulatory T cells in the intestine through interleukin-2. Nature.

[15] Mitsialis V, Wall S, Liu P, Ordovas-Montanes J, Parmet T, Vukovic M, Spencer D (2020). Single-cell analyses of colon and blood reveal distinct immune cell signatures of ulcerative colitis and crohn’s disease. Gastroenterology.

[16] Ogino T, Nishimura J, Barman S, Kayama H, Uematsu S, Okuzaki D, Osawa H (2013). Increased Th17-inducing activity of CD14+ CD163low myeloid cells in intestinal lamina propria of patients with crohn’s disease. Gastroenterology.

[17] Corridoni D, Antanaviciute A, Gupta T, Fawkner-Corbett D, Aulicino A, Jagielowicz M, Parikh K (2020). Single-cell atlas of colonic CD8+ T cells in ulcerative colitis. Nat Med.

[18] Boland BS, He Z, Tsai MS, Olvera JG, Omilusik KD, Duong HG, Kim ES (2020). Heterogeneity and clonal relationships of adaptive immune cells in ulcerative colitis revealed by single-cell analyses. Sci Immunol.

[19] Uzzan M, Martin JC, Mesin L, Livanos AE, Castro-Dopico T, Huang R, Petralia F (2022). Ulcerative colitis is characterized by a plasmablast-skewed humoral response associated with disease activity. Nat Med.

[20] Li K, Strauss R, Ouahed J, Chan D, Telesco SE, Shouval DS, Canavan JB (2018). Molecular comparison of adult and pediatric ulcerative colitis indicates broad similarity of molecular pathways in disease tissue. J Pediatr Gastroenterol Nutr.

[21] Vancamelbeke M, Vanuytsel T, Farré R, Verstockt S, Ferrante M, Van Assche G, Rutgeerts P (2017). Genetic and transcriptomic bases of intestinal epithelial barrier dysfunction in inflammatory bowel disease. Inflamm Bowel Dis.

[22] Parigi SM, Larsson L, Das S, Ramirez Flores RO, Frede A, Tripathi KP, Diaz OE (2022). The spatial transcriptomic landscape of the healing mouse intestine following damage. Nat Commun.

[23] Stuart T, Butler A, Hoffman P, Hafemeister C, Papalexi E, Mauck Iii WM, Hao Y (2019). Comprehensive integration of single-cell data. Cell.

[24] Fu Y, Guo Z, Wang Y, Zhang H, Zhang F, Xu Z, Shen X (2022). Single-nucleus RNA sequencing reveals the shared mechanisms inducing cognitive impairment between COVID-19 and Alzheimer’s disease. Front Immunol.

[25] Morabito S, Reese F, Rahimzadeh N, Miyoshi E, Swarup V. High dimensional co-expression networks enable discovery of transcriptomic drivers in complex biological systems.
*
BioRxiv
* 2022, doi: https://doi.org/10.1101/2022.09.22.509094. https://doi.org/10.1101/2022.09.22.509094.

[26] Morabito S, Miyoshi E, Michael N, Shahin S, Martini AC, Head E, Silva J (2021). Single-nucleus chromatin accessibility and transcriptomic characterization of Alzheimer’s disease. Nat Genet.

[27] Trapnell C, Cacchiarelli D, Grimsby J, Pokharel P, Li S, Morse M, Lennon NJ (2014). The dynamics and regulators of cell fate decisions are revealed by pseudotemporal ordering of single cells. Nat Biotechnol.

[28] Qiu X, Hill A, Packer J, Lin D, Ma YA, Trapnell C (2017). Single-cell mRNA quantification and differential analysis with Census. Nat Methods.

[29] Jin S, Guerrero-Juarez CF, Zhang L, Chang I, Ramos R, Kuan CH, Myung P (2021). Inference and analysis of cell-cell communication using CellChat. Nat Commun.

[30] Mo S, Shen X, Wang Y, Liu YP, Sugasawa T, Yang ZC, Gu W (2023). Systematic single-cell dissecting reveals heterogeneous oncofetal reprogramming in the tumor microenvironment of gastric cancer. Hum Cell.

[31] Franz M, Rodriguez H, Lopes C, Zuberi K, Montojo J, Bader GD, Morris Q (2018). GeneMANIA update 2018. Nucleic Acids Res.

[32] Mostafavi S, Ray D, Warde-Farley D, Grouios C, Morris Q (2008). GeneMANIA: a real-time multiple association network integration algorithm for predicting gene function. Genome Biol.

[33] Yu G, Wang LG, Han Y, He QY (2012). clusterProfiler: an R package for comparing biological themes among gene clusters. OMICS-J Integrative Biol.

[34] Zhou Y, Zhou B, Pache L, Chang M, Khodabakhshi AH, Tanaseichuk O, Benner C (2019). Metascape provides a biologist-oriented resource for the analysis of systems-level datasets. Nat Commun.

[35] Moffitt RA, Marayati R, Flate EL, Volmar KE, Loeza SGH, Hoadley KA, Rashid NU (2015). Virtual microdissection identifies distinct tumor- and stroma-specific subtypes of pancreatic ductal adenocarcinoma. Nat Genet.

[36] Brunet JP, Tamayo P, Golub TR, Mesirov JP (2004). Metagenes and molecular pattern discovery using matrix factorization. Proc Natl Acad Sci USA.

[37] Mo S, Dai L, Wang Y, Song B, Yang Z, Gu W. Comprehensive analysis of the systemic transcriptomic alternations and inflammatory response during the occurrence and progress of COVID-19.
*
Oxid Med Cell Longev
* 2021, 2021: 9998697. https://doi.org/10.1155/2021/9998697.

[38] Subramanian A, Tamayo P, Mootha VK, Mukherjee S, Ebert BL, Gillette MA, Paulovich A (2005). Gene set enrichment analysis: a knowledge-based approach for interpreting genome-wide expression profiles. Proc Natl Acad Sci USA.

[39] Becht E, Giraldo NA, Lacroix L, Buttard B, Elarouci N, Petitprez F, Selves J (2016). Estimating the population abundance of tissue-infiltrating immune and stromal cell populations using gene expression. Genome Biol.

[40] Kuppe C, Ramirez Flores RO, Li Z, Hayat S, Levinson RT, Liao X, Hannani MT (2022). Spatial multi-omic map of human myocardial infarction. Nature.

[41] Tibshirani R. Regression shrinkage and selection via the Lasso.
*
J R Stat Soc B
* 1996, 58: 267–288. https://doi.org/10.1111/j.2517-6161.1996.tb02080.x.

[42] Lang M, Binder M, Richter J, Schratz P, Pfisterer F, Coors S, Au Q,
*et al*. mlr3: a modern object-oriented machine learning framework in R.
*
Journal of Open Source Software
* 2019, 4: 1903. https://doi.org/10.21105/joss.01903.

[43] Wang Z, Li S, Cao Y, Tian X, Zeng R, Liao DF, Cao D (2016). Oxidative stress and carbonyl lesions in ulcerative colitis and associated colorectal cancer. Oxid Med Cell Longev.

[44] Shen Y, Ma J, Yan R, Ling H, Li X, Yang W, Gao J (2015). Impaired self-renewal and increased colitis and dysplastic lesions in colonic mucosa of AKR1B8-deficient mice. Clin Cancer Res.

[45] Jena G, Trivedi PP, Sandala B (2012). Oxidative stress in ulcerative colitis: an old concept but a new concern. Free Radical Res.

[46] Shen X, Mo S, Wang Y, Lin L, Liu Y, Weng M, Gu W (2023). Single‐cell dissection reveals the role of DNA damage response patterns in tumor microenvironment components contributing to colorectal cancer progression and immunotherapy. Genes Cells.

[47] Catana C, Magdas C, Tabaran F, Crăciun E, Deak G, Magdaş V, Cozma V (2018). Comparison of two models of inflammatory bowel disease in rats. Adv Clin Exp Med.

[48] Barrow F, Khan S, Wang H, Revelo XS (2021). The emerging role of B cells in the pathogenesis of NAFLD. Hepatology.

[49] Perl A (2013). Oxidative stress in the pathology and treatment of systemic lupus erythematosus. Nat Rev Rheumatol.

[50] Poznyak AV, Bezsonov EE, Popkova TV, Starodubova AV, Orekhov AN (2021). Immunity in atherosclerosis: focusing on T and B cells. Int J Mol Sci.

[51] Erikson E, Ádori M, Khoenkhoen S, Zhang J, Rorbach J, Castro Dopico X, Karlsson Hedestam G (2022). Impaired plasma cell differentiation associates with increased oxidative metabolism in IκBNS-deficient B cells. Cell Immunol.

[52] Kaenkumchorn T, Wahbeh G (2020). Ulcerative colitis. Gastroenterol Clin N Am.

[53] Lu J, Wang Z, Maimaiti M, Hui W, Abudourexiti A, Gao F (2022). Identification of diagnostic signatures in ulcerative colitis patients via bioinformatic analysis integrated with machine learning. Hum Cell.

[54] Mayr JA, Merkel O, Kohlwein SD, Gebhardt BR, Böhles H, Fötschl U, Koch J (2007). Mitochondrial phosphate–carrier deficiency: a novel disorder of oxidative phosphorylation. Am J Hum Genet.

[55] Zhang D, Yan P, Han T, Cheng X, Li J (2021). Identification of key genes and biological processes contributing to colitis associated dysplasia in ulcerative colitis. PeerJ.

[56] Liu Y, Adachi M, Zhao S, Hareyama M, Koong AC, Luo D, Rando TA (2009). Preventing oxidative stress: a new role for XBP1. Cell Death Differ.

[57] Kaser A, Lee AH, Franke A, Glickman JN, Zeissig S, Tilg H, Nieuwenhuis EES (2008). XBP1 links ER stress to intestinal inflammation and confers genetic risk for human inflammatory bowel disease. Cell.

[58] Han NY, Choi W, Park JM, Kim EH, Lee H, Hahm KB (2013). Label-free quantification for discovering novel biomarkers in the diagnosis and assessment of disease activity in inflammatory bowel disease. J Digestive Dis.

[59] Shetty GA, Hattiangady B, Upadhya D, Bates A, Attaluri S, Shuai B, Kodali M (2017). Chronic oxidative stress, mitochondrial dysfunction, Nrf2 activation and inflammation in the hippocampus accompany heightened systemic inflammation and oxidative stress in an animal model of gulf war illness. Front Mol Neurosci.

[60] Xu J, Li D, Lu Y, Zheng TY (2021). Aβ monomers protect lens epithelial cells against oxidative stress by upregulating CDC25B. Free Radical Biol Med.

[61] Pandey S, Sharma A, Tripathi D, Kumar A, Khubaib M, Bhuwan M, Chaudhuri TK,
*et al*.
*Mycobacterium tuberculosis* peptidyl-prolyl isomerases also exhibit chaperone like activity
*in-vitro* and
*in-vivo*.
*
PloS One
* 2016, 11: e0150288. https://doi.org/10.1371/journal.pone.0150288.

[62] Woo IS, Eun SY, Jang HS, Kang ES, Kim GH, Kim HJ, Lee JH (2009). Identification of ADP-ribosylation factor 4 as a suppressor of N-(4-hydroxyphenyl)retinamide-induced cell death. Cancer Lett.

[63] Zhang B, Li X, Liu G, Zhang C, Zhang X, Shen Q, Sun G (2021). Peroxiredomin-4 ameliorates lipotoxicity-induced oxidative stress and apoptosis in diabetic cardiomyopathy. Biomed Pharmacother.

[64] Chen Z, Li H, Yang T, Chen T, Dong C, Gu Q, Cheng X (2020). Transcriptome analysis provides insights into the molecular bases in response to different nitrogen forms-induced oxidative stress in tea plant roots (Camellia sinensis). Funct Plant Biol.

[65] Bergemalm D, Andersson E, Hultdin J, Eriksson C, Rush ST, Kalla R, Adams AT (2021). Systemic inflammation in preclinical ulcerative colitis. Gastroenterology.

