# Supplementary material for: Single-cell dissection, hdWGCNA and deep learning reveal the role of oxidatively stressed plasma cells in ulcerative colitis: Oxidative stress in plasma cells and ulcerative colitis
Source: Acta Biochim Biophys Sin (Shanghai). 2023 Oct 9;55(11):1730–9. doi: 10.3724/abbs.2023237 (PMC10686794; doi:10.3724/abbs.2023237)
Supplement: Supplementary [file Supplementary.pdf]

Single-cell dissecting, hdWGCNA and deep learning revealed the role of oxidatively stressed plasma cells in ulcerative colitis

Supplementary Table 1-2

Supplementary Figure 1

Table S1. Genes in the blue module

gene_name	module	kME
FTL	blue	0.38133
SELT	blue	0.38251
PSMB2	blue	0.38309
IDH2	blue	0.38376
PSMD8	blue	0.38409
ATP6V0B	blue	0.38485
DERL1	blue	0.38624
TMED9	blue	0.38687
LDHA	blue	0.38788
SUMO2	blue	0.38992
ZNF706	blue	0.39398
SELK	blue	0.39404
NDUFB11	blue	0.39455
TXNDC15	blue	0.39632
ERGIC3	blue	0.39748
CUTA	blue	0.39766
EDF1	blue	0.39788
NDUFV2	blue	0.39828
PARK7	blue	0.39979
AURKAIP1	blue	0.40002
PRELID1	blue	0.40074
TMBIM6	blue	0.40102
PPA1	blue	0.4011
PSMA7	blue	0.40161
LMAN2	blue	0.40237
ATP5O	blue	0.40252
EIF3E	blue	0.40502
PSMB1	blue	0.40619
ITM2C	blue	0.40673
GUK1	blue	0.40681
LDHB	blue	0.40869
MRPL51	blue	0.41239
SLC25A3	blue	0.41254
CFL1	blue	0.41298
CYCS	blue	0.41304
CYBA	blue	0.41474
ARF4	blue	0.41532
COX8A	blue	0.41674
COX6A1	blue	0.41755
NDUFAB1	blue	0.41762
LMAN1	blue	0.41853

PSMB6	blue	0.42093
PSME1	blue	0.42119
POMP	blue	0.42152
PRDX5	blue	0.4242
ATP5A1	blue	0.42551
SRP14	blue	0.42583
SLC25A5	blue	0.42651
GLRX	blue	0.42894
EIF3K	blue	0.42919
H2AFZ	blue	0.4338
UQCRH	blue	0.43693
ATP5J	blue	0.43761
ARPC3	blue	0.44014
YBX1	blue	0.44317
DNAJB11	blue	0.44336
JTB	blue	0.44601
PPIA	blue	0.44656
RAN	blue	0.44732
SPCS3	blue	0.45015
ARPC2	blue	0.45043
DAD1	blue	0.45473
COX5A	blue	0.45598
COPE	blue	0.45767
XBP1	blue	0.45794
H3F3A	blue	0.45855
PSME2	blue	0.45967
SSR2	blue	0.46274
PRDX4	blue	0.46581
SEC61B	blue	0.46947
GSTP1	blue	0.47098
MTDH	blue	0.47628
SERP1	blue	0.47915
RPL22L1	blue	0.48043
TMED2	blue	0.49007
RABAC1	blue	0.49398
COX7A2	blue	0.50392
NDUFA4	blue	0.50523
MYL6	blue	0.5078
OSTC	blue	0.50922
SDF2L1	blue	0.51256
ATP5G3	blue	0.51599
EIF1	blue	0.51962
SPCS2	blue	0.52695
SPCS1	blue	0.5297

ISG20	blue	0.53232
SSR3	blue	0.54221
EEF1B2	blue	0.54821
OAZ1	blue	0.5522
MANF	blue	0.55342
PPIB	blue	0.57204
FKBP11	blue	0.57392
RPL7A	blue	0.58075
FTH1	blue	0.5892
RPLP0	blue	0.59551
CHCHD2	blue	0.59775
SUB1	blue	0.61558
MYDGF	blue	0.61961
SEC11C	blue	0.62318
MZB1	blue	0.6489

Table S2. Genes selected by LASSO regression

Gene	Coef
SLC25A3	-1.424671731
XBP1	0.173510822
PSME1	0.845557231
COX6A1	-1.609336135
PPIB	3.085500772
ARF4	2.193092359
PRDX4	2.690146165
NDUFAB1	-3.937018246
TMED9	3.358007725
MYL6	-5.107412525
SPCS3	2.120320231

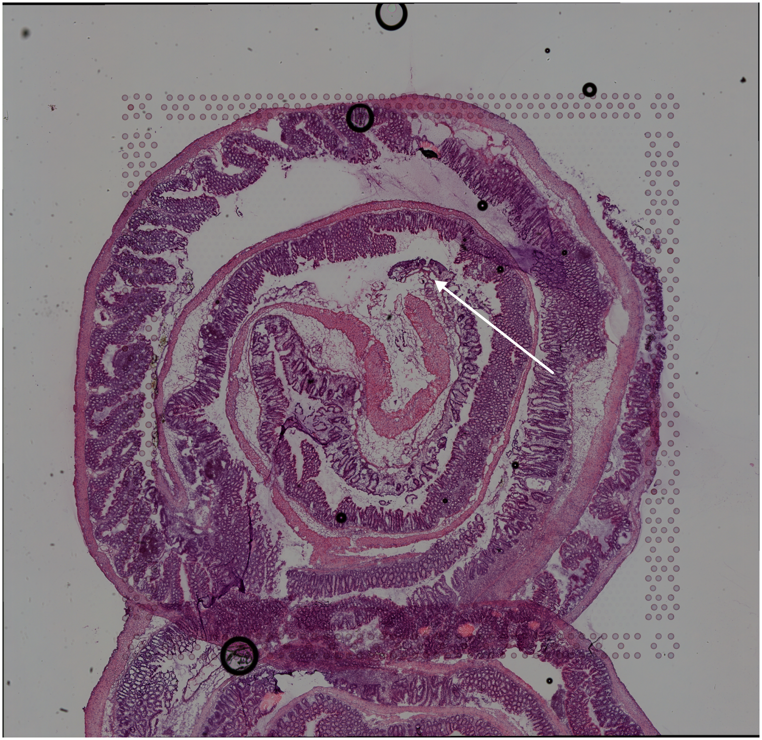

Supplementary Figure 1. Hematoxylin-eosin staining of colon of a DSS colitis mouse
